# Treatment of Facioscapulohumeral Muscular Dystrophy (FSHD): A Systematic Review

**DOI:** 10.7759/cureus.39903

**Published:** 2023-06-03

**Authors:** Alex S Aguirre, Olga M Astudillo Moncayo, Johanna Mosquera, Veronica E Muyolema Arce, Camila Gallegos, Juan Fernando Ortiz, Andres F Andrade, Sebastian Oña, Maja J Buj

**Affiliations:** 1 School of Medicine, Universidad San Francisco de Quito, Quito, ECU; 2 General Medicine, University of Cuenca, Cuenca, ECU; 3 Medicine, Universidad de las Américas, Quito, ECU; 4 Medicine, Universidad de Guayaquil, Guayaquil, ECU; 5 Colegio de Ciencias de la Salud, Universidad San Francisco de Quito, Quito, ECU; 6 Neurology, Spectrum Health Medical Group/Michigan State University, Quito, ECU; 7 Medicine, Universidad San Francisco de Quito, Quito, ECU; 8 Psychiatry, Medical Chamber, Belgrade, USA

**Keywords:** zinc., vitamin e, vitamin c, myo029, diltiazem, losmapimod, salbutamol, albuterol, facioscapulohumeral muscular dystrophy

## Abstract

Facioscapulohumeral muscular dystrophy (FSHD) is the third most common type of muscular dystrophy. This disease presents as a slowly progressive asymmetric muscle weakness that involves the facial, scapular, and upper arm muscles mainly. Currently, there is no established consensus on this disease treatment in terms of medications. We assessed the response to the treatment of the drugs utilized in clinical trials by performing a systematic literature review in English using the preferred reporting items for systematic reviews (PRISMA) and meta-analyses. We only used human clinical trials in patients diagnosed with FSHD that received consistent pharmacological treatment. We included 11 clinical trials that fulfilled our criteria. We concluded that albuterol had statistically significant results in three out of four clinical trials, with improved elbow flexors muscle strength. Vitamin C, vitamin E, zinc gluconate, and selenomethionine showed significant improvement in the maximal voluntary contraction and endurance limit time of quadriceps muscle. At the same time, diltiazem and MYO-029 demonstrate no improvement in function, strength, or muscle mass. Losmapimod, currently in phase I of the ReDUX4 trial, showed promising results. Peradventure, more clinical trials are still needed to address this subject. Nevertheless, this review provides a clear and concise update on the treatment for this disease.

## Introduction and background

Facioscapulohumeral muscular dystrophy (FSHD) is the third most common muscular dystrophy after muscular dystrophy of Duchenne and myotonic dystrophy. In the United States, the prevalence is one in 15,000-21,000 individuals. The overall prevalence of this disorder is 4-12 in 100,000 individuals [1]. The disease presentation varies greatly between patients; however, it commonly starts with asymmetric muscle involvement of the facial muscles, being evident signs like incomplete eye closure or a flat smile. Then, the disease progresses to proximal muscles in the upper trunk like the rhomboids and the anterior serratus that can present with winging scapula or inability to adduct the arm more than 90°. Finally, the distal muscles of the lower extremities and more proximal muscles become affected. As FSHD progresses, specific signs like increased axillary fat, and proximal arm muscle wasting become prevalent. The forearm is usually spared, which causes the Popeye or Beevor sign, characteristically associated with this dystrophy [2].

The deletion of significant repeated elements of the long arm of the subtelomeric region of chromosome 4q, known as D4Z4 of the gen DUX4, appears to explain FSHD [2]. First, a deletion in the D4Z4 region occurs; the DNA of this region is methylated in transcriptionally silent heterochromatin. Because of this deletion, a more permissive chromatin structure developed, allowing the transcription of pathologic proteins that result in disease. The clinical presentation correlates with the smallest number of residual D4Z4 units [3].

The DUX4 is overexpressed, as mentioned before, and when DUX4 is overexpressed is highly toxic, causing caspase-3-mediated apoptosis, causing a negative effect on myogenesis. In addition, activation of the DUX4 gene involves proteins that cause atrophy and protein degradation [4]. Finally, DUX4 also activates protein of the innate immune system which could explain why there are inflammatory infiltrates mainly in the perivascular region [4].

The inheritance pattern of this disease is mainly autosomal dominant with a slowly progressive clinical course [4]. As life-threatening clinical manifestations are rare, life expectancy is unaffected. The disease usually occurs in adolescence but can present at earlier stages of life [5].

For diagnosis, clinical and genetic confirmation can be done if there is a high suspicion. The current treatment for the disease is mainly supportive, which makes this review a valuable tool to guide and develop future therapies. Table 1 shows current therapies for FSHD patients [6].

**Table 1:** Current therapies for FSHD patients. FSHD, facioscapulohumeral muscular dystrophy

Current therapies	Specifics
Physical therapy	Varies depending on the stage of the disease.
Exercise	Recommended 30 min, three times a day (data are limited regarding the efficacy of the exercise).
Orthopedic intervention	Deltoid muscles are usually preserved, and these patients benefit from scapulothoracic fusion which may improve abduction and flexion of the arm.
Pain management	There are only a few numbers of studies addressing pain management nowadays. Current pain management consists of NSAIDs and physical therapy, which is the main aid for this common problem in FSHD patients.
Respiratory Surveillance	Respiratory insufficiency is rare in FSHD patients, pulmonary function and monitors the forced vital capacity routinely, especially in patients with severe proximal weakness, thoracic cage bony abnormalities, and any patients with other pulmonary or cardiac problems. Respiratory insufficiency is most common while sleeping, so if any of the previously mentioned conditions are present, then nocturnal sleep monitoring is first indicated.
Eye care	A retinal exam is indicated at the time of FSHD diagnosis. Retinal vascular and telangiectatic neovascular diseases are common, and photocoagulation is an effective treatment if performed early to prevent retinal damage or detachment. Keratitis is also expected due to facial weakness and incomplete palpebral closure, which may be prevented and managed with ophthalmic patches and hydrant gels.
Hearing loss	Baseline hearing testing is essential to prevent any developmental or learning problem that may affect language or school performance, especially in infants and children.

While there have been advances in understanding the pathology and genetics of FSHD, the treatment is mainly unknown. We conducted a systematic review to investigate the drugs/medications used in clinical trials and observational studies to treat FSHD.

## Review

Materials and methods

Protocol

To conduct this systematic review, we used the following protocol: preferred reporting items for systematic reviews (PRISMA) and meta-analysis [7].

Eligibility Criteria and Study Selection

We only included clinical trials conducted on humans and written in English. Animal studies were excluded. We excluded papers that did not fulfill the aims of our research. After screening the studies, we only included papers with one of the following characteristics: (1) Patients: individuals with facioscapulohumeral dystrophy; (2) Intervention: Any medication used for the treatment of this disorder; (3) Comparator: control group or placebo; and (4) Outcomes: death, progression of the disease, quality of life.

Database and Search Strategy

We used PubMed and Google Scholar as databases. The search was conducted between 10 June 2022 and 20 June 2022. As an Advance search we used the following terms: ("facioscapulohumeral muscular dystrophy"[Title/Abstract] AND "treatment"[Title/Abstract]) OR ("facioscapulohumeral muscular dystrophy"[Title/Abstract] AND "management"[Title/Abstract]) OR ("facioscapulohumeral muscular dystrophy"[Title/Abstract] AND "therapy"[Title/Abstract]).

Data Extraction and Analysis

We collected the following information from each paper: methods, dose, duration, route of administration, number of participants, study design, and patient selection. We also extracted the main results, including each clinical trial's outcome measure and main limitations. We analyzed the primary and secondary studies’ goals and gathered the main conclusions from each study.

Bias Assessment

To assess the limitations and bias of this systematic review, we used the: Cochrane collaboration risk-of-bias [8].

Results

**Figure 1:**
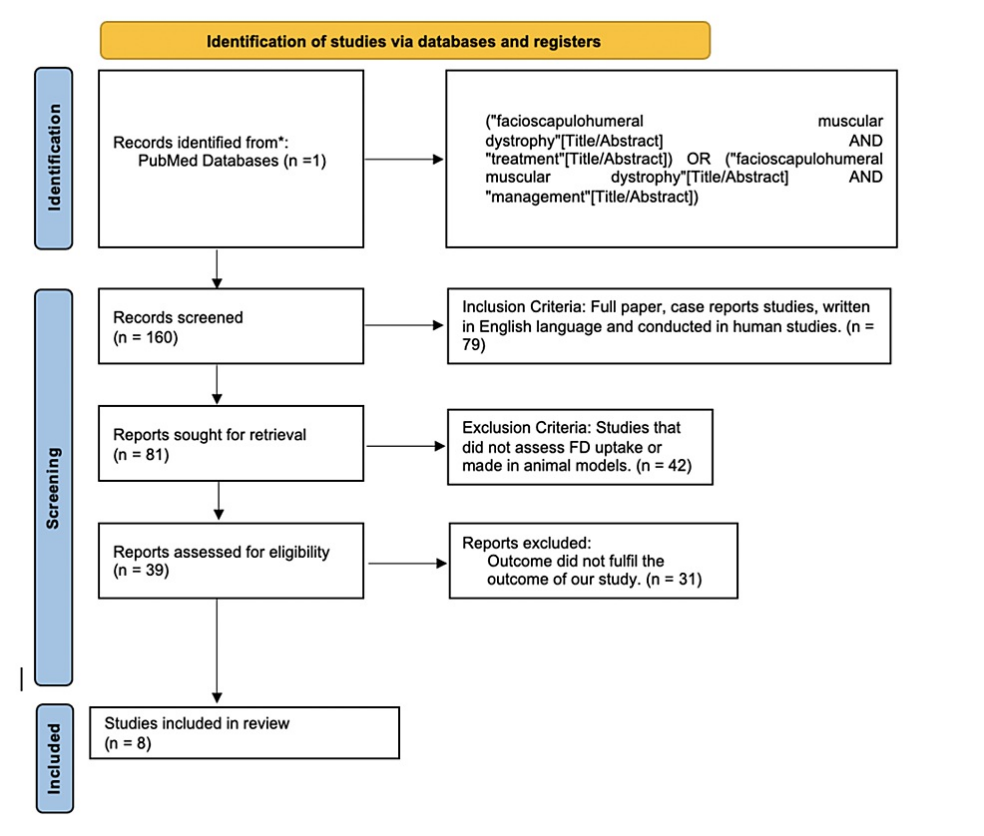
PRISMA flow chart of this systematic review. PRISMA, preferred reporting items for systematic reviews and meta-analysis

Figure 1 shows the PRISMA flow chart of the systematic review.

Study characteristics

We gathered nine articles from five different drugs: Salbutamol, Albuterol, Losmapimod, Diltiazem, 2MWT, and ACE-083. Table 2 shows the characteristics of studies: [9-17].

**Table 2:** Study characteristics of the studies. IV, intravenous; BID, twice a day; PO, oral dose; QD, every four days; BB, biceps brachii; TA, tibialis anterior; FSHD, facioscapulohumeral muscular dystrophy

Author and year of publication	Drug, Country, Study Design	No. of patients In the treatment group	No. of patients In the control group	Patient selection	Dose, duration, route of administration
Payan et al. (2009) [9]	Salbutamol, France, randomized controlled trial	56	56	Genetically confirmed FSHD, aged 18-60, ambulant	4 mg BID, 8 mg BID, all sustained release, orally for 24 weeks
van der Kooi et al. (2004) [10]	Albuterol, Netherlands, randomized double-blind, placebo-controlled trial	30	35	Genetically confirmed FSHD, aged 18-65, ambulant	8 mg sustained release BID, orally for 26 weeks
Kissel et al. (1998) [11]	Albuterol, USA, pilot trial	15	0	FSHD clinical diagnosis, aged 18-50, ambulant	4 mg sustained release BID, 8 mg BID orally for 12 weeks
Kissel et al. (2001) [12]	Albuterol, USA, randomized double-blind, placebo-controlled trial	60	30	FSHD clinical diagnosis, aged 18-60	4 mg BID, 8 mg BID, 16 mg BID, all sustained release, orally for 52 weeks
Mellion et al. (2021) [13]	Losmapimod, USA, phase 1, randomized, open-label, clinical trial	12	3	FSHD with clinical and genetic diagnosis aged 18-65. There was also confirmation with a biopsy.	Six patients received 7.5 mg of Losmapimod BID for 14 days; six patients received 15 mg of Losmapimod BID for 14 days; three patients placebo In the second part of the study; five patients with FSHD 15 mg PO BID for 14 days (the subjects in the second part had higher Ricci scores than the subjects in the first part).
Elsheikh et al. (2007) [14]	Diltiazem, USA, open-label trial	19	0	FSHD with clinical and genetic diagnosis, aged 21-60 ambulatory patients.	The dose is not mentioned. Duration: 24 weeks
Passerieux et al. (2014) [15]	Vitamin C Vitamin E Zinc gluconate Selenomethionine, France, Randomized, double-blind, placebo-controlled pilot clinical trial	25	27	Genetically confirmed, and positive family history for FSHD, aged 18-60.	The study included 25 patients randomly assigned to receive vitamin C: 500 mg, vitamin E: 400 mg, zinc gluconate: 25 mg and selenomethionine: 200 µg or 27 receiving placebos. Each one PO QD for 17 weeks.
Statland et al. (2022) [16]	ACE-083, USA, randomized phase 2, double-blinded, open-label trial	BB N-14	PLB N-15	At least 18 years of age with genetically confirmed FSHD1 or FSHD2 (or had a first-degree relative with genetically confirmed FSHD1 or FSHD2)	There were 2 phases to this trial Part 1 - open-label, uncontrolled, dose escalation every 3 weeks for up to 5 doses ACE-083 was given in the BB unilaterally at 150, 200, or 240 mg per muscle. And TA cohorts, ACE-083 was administered unilaterally at 150 or 200 mg per muscle or bilaterally at 200 mg per muscle Part 2 - double-blinded period, randomized 1:1 ACE-083 240 mg per muscle (BB or TA) every 3 weeks for 27 weeks
		TA N-14	PLB N-15		
Wagner et al. (2008) [17]	MYO-029, USA, phase I/II, multinational, randomized, double-blind, placebo-controlled, ascending dose, safety trial	30	11	FSHD with clinical and molecular diagnosis, aged 18 or over.	The study included 41 subjects divided into sequential dose-escalation cohorts, 30 receiving MYO-029 and 11 receiving placebos. Cohort 1: 1 mg/kg; Cohort 2: 3 mg/kg; Cohort 3: 10 mg/kg. Administered IV every 2 weeks for 6 months. After the start of the study, safety data from a multiple ascending dose study in healthy subjects became available, permitting to add Cohort 4: 30 mg/kg. Administered IV every 2 weeks for 6 months. Subsequently, due to the occurrence of hypersensitivity reactions and a case of unconfirmed aseptic meningitis in the 10/mg/kg group, investigators decided to terminate Cohort 4.

We gathered nine articles from five different drugs: Salbutamol, Albuterol, Losmapimod, Diltiazem, 2MWT, and ACE-083. Table 3 shows the characteristics of studies: [9-17]

**Table 3:** Outcome, results, and main conclusion of the study [9-17]. QMT, quantitative muscle testing; MMT, manual muscle testing; VAS, visual analog scale; CIS, checklist individual strength; SIP, sickness impact profile; SCL, symptom checklist-90; TMT, time motor testing; MVICT, maximum voluntary isometric contraction testing; DEXA, dual energy-x mass absorptiometry; PK, pharmacokinetics; TE, target engagement; SD, standard deviation; AUC (h*ng/mL) 0-12, area under the concentration vs. time curve at 12 h post dose; Cmax, maximum concentration; pHSP27:HSP27, phosphorylated heat shock protein 27:total heat shock protein 27; AMS, average muscle score; 2-MWT, two-minute walking test; MVCQD, maximal voluntary contraction; MVCQND, maximal voluntary endurance; TlimQD, limit time of the dominant quadriceps; TlimQND, limit time of the non-dominant quadriceps; TMV, total muscle volume; FF, fat fraction; CMV, contractile muscle volume; BB, performance of upper limb midlevel/elbow score; TA, functional measures included a 6-min walk test, 10-m walk/run, and four-stair climb

Author, year, drug	Drug: outcome	Results of treatment group	Results of Control or placebo group	Main conclusion
Payan et al. (2009) [9]	Salbutamol: QMT, MMT, TM	QMT: In the treatment group the QMT changes from the baseline: 2.34 ± 1.07 to 0.08 ± 0.26 in the upper limbs after 6 months (p = 0.11). While in the lower limbs the change is from 2.19 ± 1.45 to -0.01 ± 0.49 (p = 0.48)	QMT: In the placebo group the QMT changes from the baseline: 2.32 ± 1.19 to 0.02 ± 0.31 in the upper limbs after 6 months (p = 0.11) While in the lower limbs the change is from 2.32 ± 1.15 to -0.04 ± 0.36 (p=0.48)	Salbutamol did not prove benefit over placebo. This was the first multicenter study.
van der Kooi et al. (2004) [10]	Albuterol: VAS, CIS, SIP, SCL, MVIC	MMT: In the treatment group the MMT changes from the baseline: 3.35 ± 0.67 to 0.04 ± 0.21 in the upper limbs after 6 months (p = 0.58). While in the lower limbs the change is from 3.94 ± 0.87 to 0.05 ± 0.20 (p = 0.78) VAS: In the treatment group the VAS change from baseline of 10.5 (1.6-19.3) to 12.0 (3.1-20.9) after 52 weeks. CIS: In the treatment group the CIS change from baseline of 30.5 (24.3-36.6) to 28.0 (22.1-33.9) after 52 weeks. SIP: In the treatment group the SIP change is from baseline of 723 (402-1,043) to 599 (247-951) after 52 weeks. In the treatment group the MVIC change is from the baseline: 14.0 (10.2-17.8) to 14.1 (10.3- 17.9) in the elbow flexion right after 52 weeks. While in the ankle dorsiflexion right change from 9.9 (6.7-13.1) to 8.0 (4.8-11.2).	MMT: In the placebo group the MMT changes from the baseline: 3.13 ± 0.62 to 0.03 ± 0.18 in the upper limbs after 6 months (p = 0.58). While in the lower limbs the change is from 3.74 ± 0.79 to 0.04 ± 0.19 (p = 0.78) VAS: In the placebo group the VAS change is from a baseline of 13.4 (4.9-22.0) to 9.0 (0.4-17.6) after 52 weeks. CIS: In the placebo group the CIS change is from a baseline of 27.1 (21.1- 33.0) to 26.1 (20.0-31.8) after 52 weeks. SIP: In the placebo group the SIP change is from a baseline of 691 (380- 1,001) to 565 (227-903) after 52 weeks. Albuterol MVIC In the treatment group the MVIC changes from the baseline: 14.0 (10.2-17.8) to 14.1 (10.3-17.9) in the elbow flexion right after 52 weeks. While in the ankle dorsiflexion right change from 9.9 (6.7-13.1) to 8.0 (4.8-11.2). In the placebo group the MVIC changes from the baseline: 17.3 (13.7- 21.0) to 16.0 (12.4-19.7) in the elbow flexion right after 52 weeks. While in the ankle dorsiflexion right change from 10.8 (7.8-13.9) to 9.6 (6.5-12.6). Albuterol showed a short-term anabolic effect, but did not prevent long-term effects	Albuterol, nor strength training showed any benefit on pain, psychological distress, experienced fatigue, or functional status.
Kissel et al. (1998) [11]	Albuterol: DEXA, MVICT	DEXA: The DEXA had a mean change of 1.29 ± 1.81 after 12 weeks. MVICT: The MVICT had a mean change of 0.33 ± 0.60 after 12 weeks.	No control group	Albuterol did improve muscle mass and strength
Kissel et al (2001) [12]	Albuterol: MV ICT, MMT	MVICT: Low dose -0.004 ± 0.84; high dose group 0.08 ± 0.98 MMT: low dose - 0.003 ± 0.13; high dose 0 ± 0.15	MVICT: 0.20 ± 0.91 MMT: 0.04 ± 0.16	Albuterol did not improve global strength or function in patients with FSHD, nevertheless, it increased muscle mass (DEXA (+1.57 ± 1.71 kg) compared to placebo (0.25 ± 2.24; p = 0.007) and improved some measures of strength.
Mellion et al. (2021) [13]	Losmapimod: PK, TE	In subjects with FSHD dosed BID for 14 days, the highest observed mean percent change from baseline pHSP27: HSP27 ratio in sorbitol-treated blood at Day 14 was an increase of 8.0% (ratio 1.08 ± 0.07) dose-dependent concentrations in muscle (42.1 ± 10.5 ng/g [7.5 mg] to 97.2 ± 22.4 ng/g [15 mg]) were observed, with plasma-to-muscle was 34.3% (95% CI, 52.6 to 9.1%) at 7.5 mg and 42.6% (95% CI, 58.2 to 21.1%) at 15 mg (p = 0.017 and p = 0.004, respectively).	Compared to placebo after the 14 days of dosing, the ratio of pHSP27:HSP27 in sorbitol-treated blood showed a mean percent change of 26.5% (95% CI, 50.7%-9.4%) and 48.6% (95% CI, 65.4%-23.9%) at 7.5 and 15 mg (p = 0.110 and p = 0.003), respectively, with the highest reduction measured approximately 3.5 h after taking Losmapimod	Losmapimod showed sustained and robust target Inhibition (p38 α/β) in blood and muscle, showing a pHSP27:HSP27 radio reduction compared to placebo; resulting in a significant reduction of DUX4-driven gene expression. In the second part of the study, the effects were less pronounced, which may be due to the fact that HSP27 levels were diminished in diseased muscle compared with healthy-appearing muscle (subjects in the second part of the study had higher Ricci scores). This study supports further investigation of Losmapimod 15 mg BID as a potential disease-modifying therapy.
Elsheikh et al., 2007 [14]	Diltiazem: MMT, MVICT, AMS	MMT: The study had a greater than 80% power to detect a mean change from a baseline of 0.13 MMT units, based on a two-tailed paired t-test carried out at a 5% level of significance and assuming an SD of 0.19. MVICT: mean change for all patients from baseline to week 24 was 0.113 ± 2.077 (n = 17; p = 0.825) AMS: mean change for all patients from baseline to week 24 was -0.0025 to ± 0.1320 (p= 0.935). MMT (average from all the muscles tested): Mean (SE) change from baseline to week 26 was: Cohort 1: 9, 3.70 (0.08) to 9, 3.73 (0.13), at 1 mg/kg. MMT (average from all the muscles tested): Mean (SE) change from baseline to week 26 was: Cohort 1: 9, 3.70 (0.08) to 9, 3.73 (0.13), at 1 mg/kg MMT (average from all the muscles tested): Mean (SE) change from baseline to week 26 was: Cohort 1: 9, 3.70 (0.08) to 9, 3.73 (0.13), at 1 mg/kg	MMT (average from all the muscles tested): Mean (SE) change from baseline to week 26 was 8, 3.70 (0.05) to 8, 3.74 (0.07).	MYO-029 administration showed no improvement in total, upper body, or lower body strength for any subgroup at any dose.
Wagner et al. (2008) [17]	MYO-029: MMT, Muscle fiber diameters	Muscle fiber diameters: Cohort 1: median -0.93% change from baseline, at 1 mg/kg. n=10. Cohort 2: median +14.4% change from baseline, at 3 mg/kg. n=7. Cohort 3: median + 15.2% change from baseline, at 10 mg/kg. n=2.	Muscle fiber diameters: median +2.7% change from baseline at placebo group. n=6.	There was an increase in muscle fiber diameters in the 10 and 3 mg/kg groups compared with the 1mg/kg treatment and placebo groups.
Passerieux et al. (2014) [15]	2-MWT, MVCQD, MVCQND, TlimQD, TlimQND	2- MWT: from baseline 162.44 ± 61.27 to 170.80 ± 61.78 at week 17. n=25 MVCQD: from baseline 15.01 ± 9.99 to 16.16 ± 10.72 at week 17. n=24 TlimQD: from baseline 13:13 ± 6:56 to 16:05 ± 6:19 at week 17. n=19 TlimQND: from baseline 12:04 ± 5:59 to 14:51 ± 5:41 at week 17. n=18. Oxidized DNA: from baseline 23.70 to 19.19 at week 17. TMV measured by MRI, LS mean percentage change from baseline to day 190 in the BB group was 16.4% greater with ACE-083 vs placebo	2-MWT: from baseline 175.96 ± 65.51 to 180.23 ± 68.14 at week 17. n=27. MVCQD: from baseline 14.31 ± 12.74 to 14.33 ± 13.037 at week 17. n=27. MVCQND: from baseline 11.82 ± 11.68 to 11.52 ± 11.93 at week 17. n=26 TlimQD: from baseline 8:30 ± 4:42 to 8:12 ± 5:03 at week 17. n=17. TlimQND: from baseline 6:54 ± 4:17 to 6:35 ± 4:26 at week 17. n=16. Oxidized DNA: from baseline 19.99 to 17.71 at week 17.	The results of this trial show that supplementation with vitamin C, vitamin E (as alpha tocopherol), zinc gluconate, and selenomethionine in FSHD patients significantly improves the maximal voluntary contraction and endurance of both quadriceps by enhancing the antioxidant defenses and reducing oxidative stress.
Statland et al. (2022) [16]	ACE-083: TMV, FF, CMV	TMV measured by MRI, LS mean percentage change from baseline to day 190 in the BB group was 16.4% greater with ACE-083 vs placebo	Changes in TMV with ACE-083 treatment in the BB group remained relatively stable through day 295. The TA group, increases in TMV from baseline observed with ACE-083 during the double-blind period appeared to wane with extended treatment	ACE-083 treatment achieved relatively large mean changes (≈10%-15%) in TMV (in the targeted muscle) compared with smaller changes (≈5%) observed more widely in studies of systemic myostatin inhibitors

Discussion

Albuterol

Albuterol is a short-acting beta 2 adrenergic receptor (B2AR) agonist commonly used to relieve symptoms during asthma attacks. The reasoning use of this drug for this disease is based on the pharmacological mechanism in the skeletal muscle. Stimulation of B2AR stimulates protein synthesis and decreases protein degradation; all these outcomes are related to the activation of the Gs pathway, which results in increased cAMP production [18]. Other pathways involved in muscle hypertrophy, such as the phosphoinositol 3 kinase (PI3K), protein kinase B (Akt), mammalian target of rapamycin (mTOR), and forkhead box-O (FOXO) pathways, are also involved [19].

We found four articles related to FSH and this drug. Three articles showed statistically significant results, van der Kooi et al. in 2004 showed a significant increase in the elbow flexors MVIC strength in the treatment group compared to the placebo group, and Kissel et al. in 1998 showed a significant increase in DEXA lean body mass in the treatment group compared to the placebo group, Kissel et al. in 2001 showed a significant increase in DEXA lean body mass in the treatment group compared to the placebo group, on the other hand, The four articles of Albuterol did not show any significant results in any of their outcomes [9-12].

The drug was well tolerated in all the studies. The major side effects reported were nervousness, tremors, cramps, and headache. The minor side effects reported were flushing, dizziness, insomnia, nausea, and vomiting.

Losmapimod

We found only one phase I study with Losmapimod for FSHD. This drug selectively inhibits p38α/β mitogen-activated protein kinases (MAPKs), which are enzymatic modulators of DUX4 expression and mediators of inflammation [14]. By inhibiting the expression of DUX4, Losmapimod reduces apoptosis in multiple cell lines in FSHD patients. The negative effect on myotubule differentiation is minimal. This study showed promising results with excellent tolerability and safety. The adverse effects were minimum and non-severe, such as headache, dizziness, somnolence, and gastrointestinal distress [14]. 

This study opened the possibility for a phase II trial, the ReDUX4 trial. That study ended, and the official results are still pending. However, Fulcrum Therapeutics, who designed the drug, exposed positive outcomes such as a slower progression of the disease and improvement in quality of life [13]. M Yet, their primary outcome, a decreased expression of DUX4, showed no difference from baseline. This finding does not necessarily mean that the drug is not working but that the method used to detect the change may not be identifying the change [20].

Diltiazem

Diltiazem is a calcium channel blocker commonly used in hypertension. We found one study with diltiazem as a possible treatment for FSHD [21]. Abnormal calcium signaling was proposed as a possible factor contributing to muscular fiber damage in many muscular dystrophies because it can alter the normal mitochondrial functions and activate anomalous concentrations of tumor necrosis factor [21]. This drug did not significantly improve the study's function, strength, or muscle mass. No adverse effects were reported. The study indicated there was no role for diltiazem in FSHD; however, further studies may be necessary to elucidate this question [21].

Vitamin C/Vitamin E/Zinc Gluconate/Selenomethionine

We found one study using vitamins and micronutrients as supplementation in patients with FSHD. These nutritional aids enhance antioxidant mechanisms by diminishing oxidative stress, which plays a crucial role in developing muscular dystrophy. This study shows a significant improvement in the maximal voluntary contraction and the endurance limit time of both quadriceps (dominant and non-dominant) from baseline and between groups. Also, this study shows that oxidative stress biomarkers were significantly lower in the treatment group. Nevertheless, significant changes in the primary outcome (two-minute walking test) were accomplished only from baseline in the treatment group but not in between groups. The authors believe these results might be due to the heterogeneity of the group and the higher number of patients with steppage gait in the treatment group. No adverse effects were reported in this study. It may be inferred that antioxidant supplementation could be helpful for patients with FSHD [15].

ACE-083

We found one phase II study using ACE-083, a recombinant fusion protein that traps primarily myostatin and activin, ligands of the transforming growth factor (TGF)-β superfamily that inhibits skeletal muscle growth and regeneration. This study was designed to identify the safety and tolerability achieved, while the efficacy is not fully understood yet. The study demonstrated statistically significant increases in TMV and CMV increased in both groups. FF only decreased in the TA group. However, these measurements did not show consistent improvements in functional or patient-reported outcomes. This data resulted in the discontinuation of the ACE- 083 development program. The most common adverse events were mild or moderate injection-site reactions. In the future, further research may be needed to fully understand the relationship of this drug in the treatment of FSHD [16].

MYO-029

We found one study with MYO-029 for FSHD. MYO-029 is a neutralizing antibody against myostatin. The reason for developing this drug is that myostatin is an endogenous negative regulator of muscle growth and, thus, has become a novel target for many muscle diseases. This study showed no improvement in strength in any muscle group; however, it did show an increase in muscle fiber diameter. Drug-related adverse effects were identified at higher doses in hypersensitivity skin reactions such as urticaria and rash [17]. Other adverse effects such as infections, arthralgias, and pain are expected in any biological agent due to immune-mediated reactions. Besides that, this study was designed to identify the safety and tolerability achieved [17]. The short duration of the trial and the sample sizes are not optimal for identifying significant changes in strength. This may imply that further research is needed to fully understand the relationship of this drug in the treatment of FSHD.

## Conclusions

Our study provides a complete overview of the treatment alternatives for FSHD, identifying available treatments with statistically significant results and those without an effect. This review raises awareness to seek and develop viable alternatives that may enhance muscle strength in patients with this condition. Our main limitation is the use of only Pubmed and Google Scholar to develop the systematic literature review.

In conclusion, among the clinical trials reviewed, albuterol had statistically significant results in three out of four trials and showed improved muscle strength of the elbow flexors. Vitamin E, Vitamin C, zinc gluconate, and selenomethionine act as antioxidants, and one trial found improvement in the maximal voluntary contraction and endurance limit time of quadriceps muscles bilaterally. On the other hand, Diltiazem and MYO-29 had no effect on the treatment groups. An ongoing trial of Losmapimod on phase I, which reduces apoptosis, has shown interesting results. Nevertheless, more clinical trials are needed to assess this particular concern.
